# Isobavachalcone Induces Multiple Cell Death in Human Triple-Negative Breast Cancer MDA-MB-231 Cells

**DOI:** 10.3390/molecules27206787

**Published:** 2022-10-11

**Authors:** Cheng-Zhu Wu, Mei-Jia Gao, Jie Chen, Xiao-Long Sun, Ke-Yi Zhang, Yi-Qun Dai, Tao Ma, Hong-Mei Li, Yu-Xin Zhang

**Affiliations:** 1 School of Pharmacy, Bengbu Medical College, 2600 Donghai Road, Bengbu 233030, China; 2 Anhui Province Biochemical Pharmaceutical Engineering Technology Research Center, Bengbu 233030, China; 3 School of Laboratory Medicine, Bengbu Medical College, 2600 Donghai Road, Bengbu 233030, China

**Keywords:** TNBC, natural product, isobavachalcone, cell death, necroptosis, RIP3

## Abstract

Standardized treatment guidelines and effective drugs are not available for human triple-negative breast cancer (TNBC). Many efforts have recently been exerted to investigate the efficacy of natural compounds as anticancer agents owing to their low toxicity. However, no study has examined the effects of isobavachalcone (IBC) on the programmed cell death (PCD) of human triple-negative breast MDA-MB-231 cancer cells. In this study, IBC substantially inhibited the proliferation of MDA-MB-231 cells in concentration- and time-dependent manners. In addition, we found that IBC induced multiple cell death processes, such as apoptosis, necroptosis, and autophagy in MDA-MB-231 cells. The initial mechanism of IBC-mediated cell death in MDA-MB-231 cells involves the downregulation of Akt and p-Akt-473, an increase in the Bax/Bcl-2 ratio, and cleaved caspases-3 induced apoptosis; the upregulation of RIP3, p-RIP3 and MLKL induced necroptosis; as well as a simultaneous increase in LC3-II/I ratio induced autophagy. In addition, we observed that IBC induced mitochondrial dysfunction, thereby decreasing cellular ATP levels and increasing reactive oxygen species accumulation to induce PCD. These results suggest that IBC is a promising lead compound with anti-TNBC activity.

## 1. Introduction

Cancer is one of the leading causes of death in humans, and breast cancer is one of the most commonly diagnosed malignancies in women worldwide [1]. Breast cancer is classified into four types: luminal A, luminal B, human epidermal growth factor receptor-2 (HER-2) positive, and triple-negative breast cancer (TNBC); approximately 15% of breast cancer patients have TNBC [2]. Compared to the other subtypes, TNBC has the highest degree of malignancy, a poor prognosis, and easily metastasizes. In addition, TNBC does not express HER-2, an estrogen receptor (ER), and a progesterone receptor (PR) as there are no approved targets and a lack of effective drugs [3,4]. Therefore, there is a requirement to research and discover novel therapeutic targets and effective drugs to treat TNBC. 

Dysfunction of the regulatory machinery of cell death can lead to autoimmune disease or carcinogenesis [5]. Generally, cancer cells are initially sensitive to apoptosis but eventually become drug-resistant owing to defects in apoptotic signaling and the overexpression of anti-apoptotic proteins [6,7]. Therefore, the discovery and development of methods and agents that can specifically treat drug-resistant cancers are urgent tasks for protecting health and saving lives [8]. Regulated necrosis, termed as necroptosis, is a promising paradigm of programmed cell death (PCD) that can be activated when the apoptotic machinery is genetically or pathogenically defective [9]. Several factors, such as ligands of death receptors (including TNF-α, TRAIL, and Fas), viral infection, reactive oxygen species (ROS), and small molecules can initiate necroptosis [10]. Developing a class of agents that target tumor cells via induction of necroptosis may substantially improve the effectiveness of chemotherapy agents, such as 5-fluorouracil, shikonin, honokiol, and deoxypodophyllotoxin, especially in cases of drug-resistant cancers [8,11,12,13,14,15,16]. Taken together, necroptosis might be emerging as an important target in cancer treatment. 

**Figure 1:**
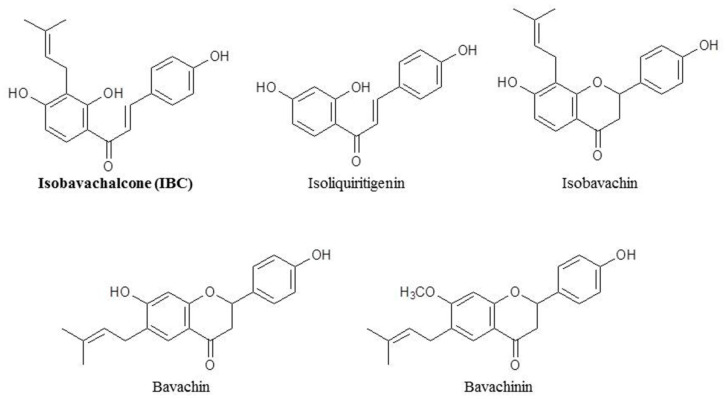
Chemical structures of isobavachalcone and its analogues.

Although treatments for breast cancer have become more diverse, chemotherapy is still one of the main strategies and means used for TNBC treatment [17]. However, conventional chemotherapy is associated with severe toxicity, and tumor cells eventually induce drug resistance. Compared with chemotherapy, natural products have many advantages, including chemical diversity, multiple targets, low toxicity, and circumvention of drug resistance [14,18,19]. Isobavachalcone (IBC), which is a member of the chalcone subclass of flavonoids isolated from the seeds of *Psoralea corylifolia* L., has various biological activities such as anti-cancer, anti-microbial, and antioxidant effects (Figure 1) [20,21,22,23]. Furthermore, IBC has various anti-cancer effects. It inhibits cell proliferation by inhibiting Akt signaling, induces ROS-dependent mitochondrial apoptosis and differentiation of HL-60 cells, induces apoptosis of human hepatocellular carcinoma HepG2 and HepG3 cells by regulating the ERK/RSK2 signaling pathway, increases E2-induced paclitaxel resistance by downregulating CD44 expression in ER-positive breast cancer cells, and induces both apoptosis and autophagy in MCF-7 cells [24,25,26,27,28]. However, the pharmacological effect and mechanism of IBC on MDA-MB-231 cell death are still unclear. 

We recently launched a screening program of necroptosis inducers from small molecules and have identified IBC from traditional Chinese medicine (TCM). In this study, we reported the effects of IBC on cell proliferation and PCD in human triple-negative breast MDA-MB-231 cells. In addition, we demonstrated that IBC induces necroptosis in MDA-MB-231 cells through effects on the RIP3-MLKL-ROS cascade.

## 2. Results

### 2.1. IBC Exhibited an In Vitro Anti-Proliferative Activity against MDA-MB-231 Cells

**Figure 2:**
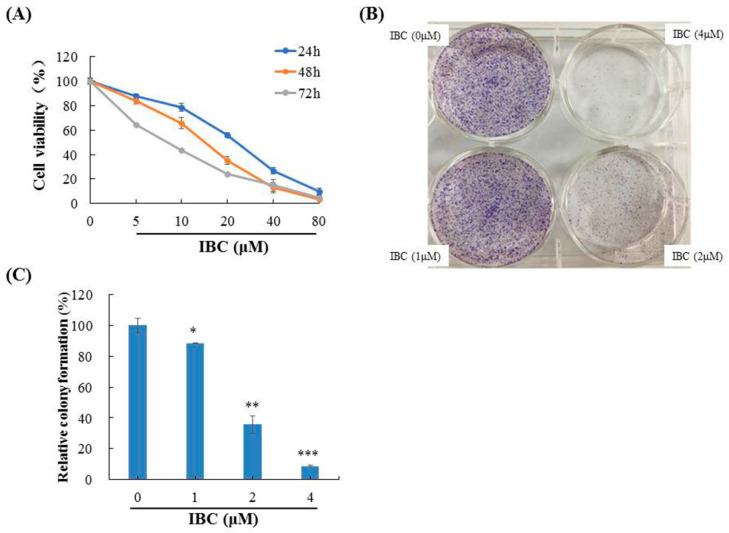
Anti-proliferative activities of IBC in MDA-MB-231 cells. (**A**) Cytotoxic activity of IBC was measured using an MTT assay. (**B**) Effects of IBC on the ability of cells to form colonies in MDA-MB-231 cells using a colony-formation assay. (**C**) Quantification of the colony-formation assay. * *p* < 0.05, ** *p* < 0.01, and *** *p* < 0.001 compared with the control.

Initially, we measured the cytotoxic activity of IBC analogs from TCM against five human cancer cell lines using an MTT assay. These results showed that IBC inhibited the growth of MDA-MB-231 cells more than other compounds (Table 1). Based on this result, IBC was selected for further investigation of its anti-cancer effect against MDA-MB-231 cells. As shown Figure 2A, the viability of human TNBC MDA-MB-231 cells gradually decreased with IBC in a concentration- and time-dependent manner, and the IC_50_ values of IBC at 24, 48, and 72 h were 21.45, 15.15, and 8.53 µM, respectively. Similar to the cell viability assay, we also observed that IBC substantially inhibited the colony-forming ability at low concentrations (Figure 2B,C). 

### 2.2. IBC Induced Apoptosis in MDA-MB-231 Cells 

**Figure 3:**
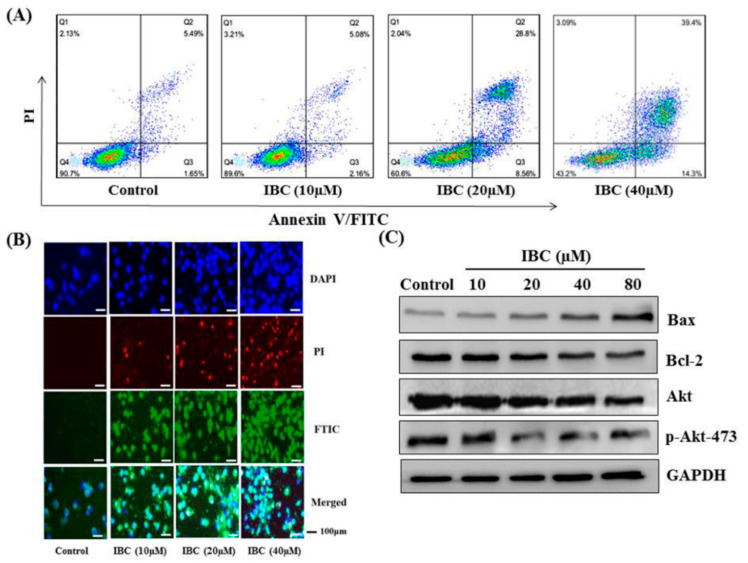
IBC induced apoptosis in human breast cancer MDA-MB-231 cells. (**A**) Flow cytometric analysis of cell death after being treated at different concentrations of IBC using annexin V-FITC/PI dual staining. (**B**) The cells were treated with IBC for 24 h, subjected to DAPI, PI, and FITC staining and visualized using fluorescence microscopy. (**C**) Western blotting analysis of Bax, Bcl-2, Akt, and p-Akt-473 protein levels.

Following incubation with IBC for 48 h, flow cytometry was used to detect cell death. Annexin V/PI dual staining resulted in cell death rates of 10.4%, 39.4%, and 56.8% for the MDA-MB-231 cell lines exposed to 10.0, 20.0, and 40.0 µM of IBC, respectively (Figure 3A). In addition, DAPI and PI staining revealed significantly increased both red and green fluorescence in the IBC exposed cells (Figure 3B). 

**Figure 4:**
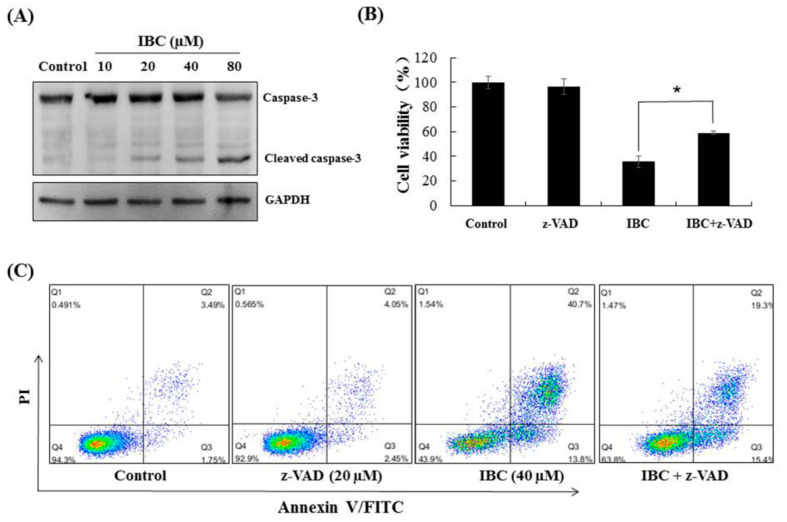
IBC induced caspase-3 dependent apoptosis in MDA-MB-231 cells. (**A**) Western blotting analysis of caspase-3. (**B**) Cell viability following treatment with IBC (40 µM) for 24 h alone or with the pan-caspase inhibitor z-VAD-FMK (20 µM) pre-treatment as measured by the MTT assay. (**C**) Annexin V-FITC/PI analysis following treatment with IBC with or without pre-treatment with z-VAD-FMK. * *p* < 0.05 compared between the groups of IBC and IBC with z-VAD-FMK.

**Figure 5:**
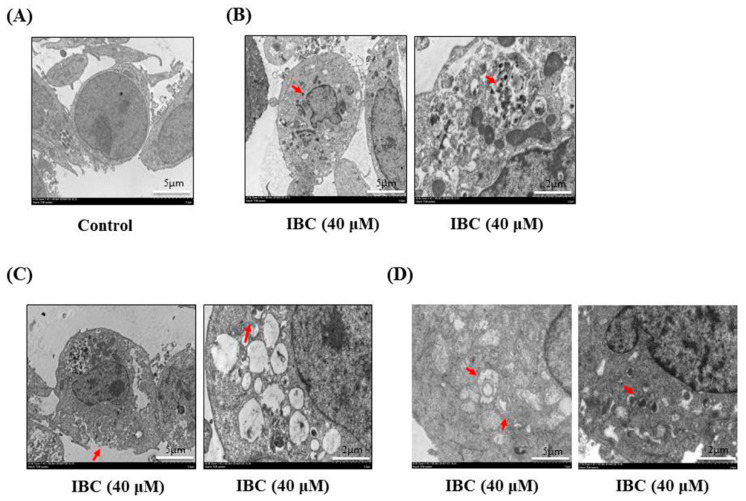
Electron microscopy of MDA-MB-231 cells treated with IBC (40 µM) for 24 h. (**A**) Morphological characteristics of control. (**B**) Morphological characteristics of IBC-treated MDA-MB-231 cells showing apoptosis. Red arrowheads indicate bright nuclear condensation. (**C**) Morphological characteristics of IBC-treated MDA-MB-231 cells showing necroptosis. Red arrowheads indicate cell membrane integrity and swelling of cellular organelles. (**D**) Morphological characteristics of IBC-treated MDA-MB- 231 cells. Red arrowheads indicate autophagosomes.

Western blot analysis showed that IBC induced apoptosis in MDA-MB-231 cells as confirmed by the downregulation of Bcl-2, Akt, and p-Akt-473, the upregulation of Bax protein levels, and cleaved caspases-3 (Figure 3C and Figure 4A). Furthermore, the MDA-MB-231 cell was pre-treated with the pan-caspase inhibitor z-VAD-FMK. The effects of IBC were decreased by treatment with z-VAD-FMK; the cell viability was 35.5% when IBC was used alone and increased to 58.9% in combination with z-VAD-FMK (*p* < 0.05) (Figure 4B). Similar to the MTT assay, we also observed that z-VAD-FMK significantly increased the viability of IBC treated MDA-MB-231 cells (Figure 4C). In addition, vehicle-treated cells showed integrated shapes and clear skeletons, whereas IBC-exposed cells displayed bright nuclear condensation and apoptotic bodies (Figure 5A,B). These results indicate that IBC induced apoptosis is caspase-dependent. 

### 2.3. IBC Induced Necroptosis in MDA-MB-231 Cells

**Figure 6:**
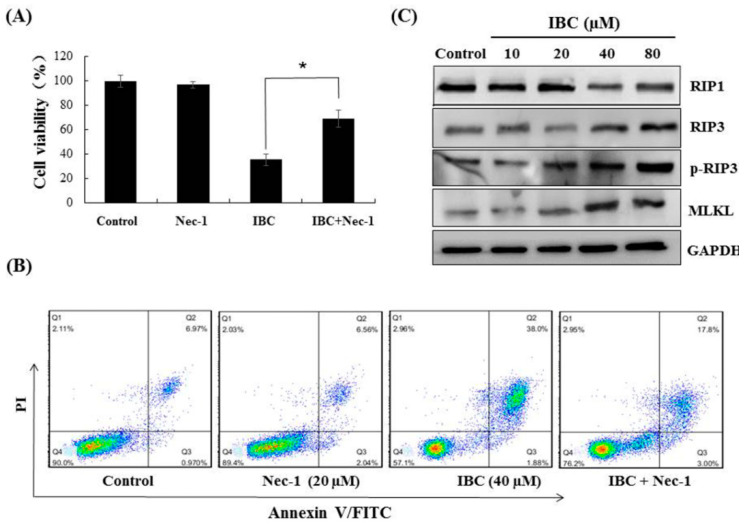

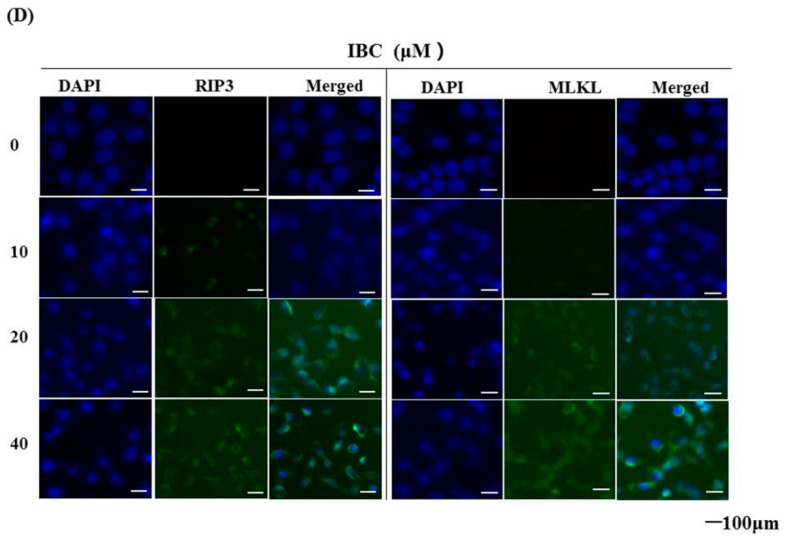
IBC induced necroptosis in human triple-negative breast MDA-MB-231 cancer cells. (**A**) Cell viability following treatment with IBC (40 µM) for 24 h alone or with the necroptosis inhibitor Nec-1 (20 µM) pre-treatment as measured by the MTT assay. (**B**) Annexin V-FITC/PI analysis following treatment with IBC with or without pre-treatment with Nec-1. (**C**) Western blotting analysis of necroptosis-related proteins (RIP1, RIP3, p-RIP3, and MLKL). (**D**) The cells were treated with IBC and incubated overnight with the primary antibodies at 4 °C. The localization of RIP3 and MLKL was assessed by immunofluorescence staining. Nuclei were stained with DAPI. * *p* < 0.05 compared between the groups of IBC and IBC with Nec-1.

Next, we observed under an electron microscope that IBC induced the morphological features of typical necrosis at the same concentration (40 µM) and time (24 h) in MDA-MB-231 cells (Figure 5C). IBC induced typical nuclear fragmentation, loss of plasma membrane integrity, and organelle swelling. Meanwhile, cell viability after pre-treatment with the necroptosis inhibitor necrostatin (Nec-1) was substantially higher than that of the cells treated with IBC (40 µM) alone, demonstrating that Nec-1 had protective effects on the IBC induced necroptosis (Figure 6A,B). RIP3 plays a critical role in the regulation of TNF-induced necroptosis, and the complex containing RIP3 functions as a “necrosome” [29]. Furthermore, we found that IBC gradually increased the expression of RIP3, p-RIP3, and MLKL (Figure 6C). In addition, IBC remarkably promoted the nuclear translocation of RIP3 and MLKL (Figure 6D).

### 2.4. Knockdown of RIP3 Using siRNA Protected MDA-MB-231 Cells against IBC Induced Necroptosis 

**Figure 7:**
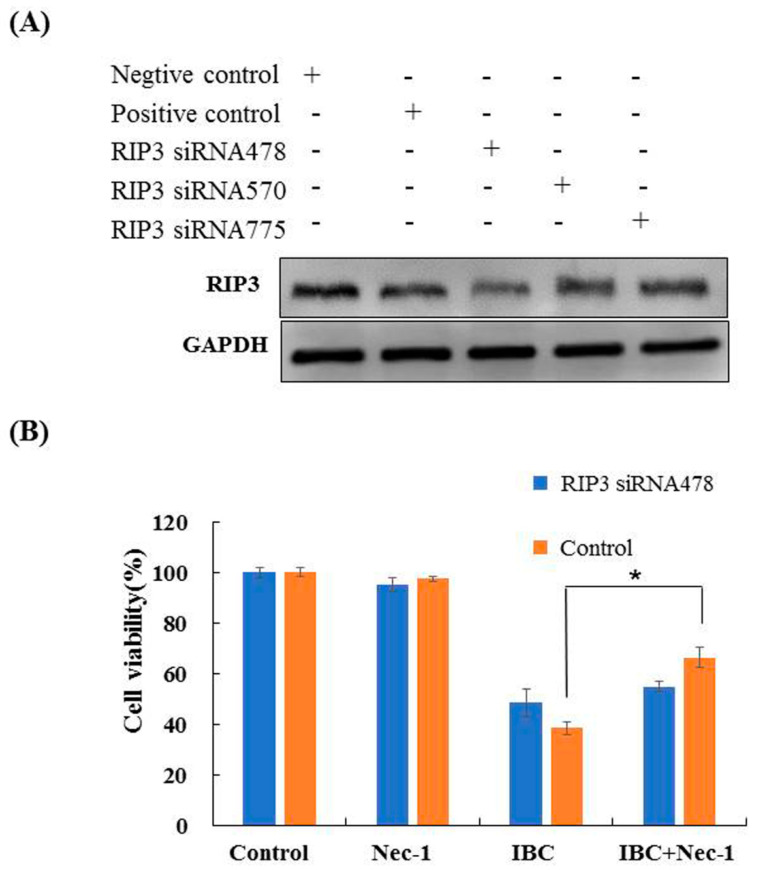
Knock-down of RIP3 with siRNA protected against IBC-induced necroptosis in MDA-MB-231 cells. (**A**) The cells were transfected with RIP3 siRNA, and whole cell lysates were subjected to Western blotting analysis. (**B**) After transfection with RIP3 siRNA, the cells were treated with IBC (40 µM) for 24 h, without or with pre-treatment with Nec-1. The cell viability was detected using the MTT assay. * *p* < 0.05 compared between the groups of IBC and IBC with Nec-1.

To explore the role of RIP3 in necroptosis, we investigated the effects of RIP3 knock- down in MDA-MB-231 cells using siRNA. The siRNA478 significantly silenced RIP3 expression, leading to a loss of Nec-1 protective ability in the IBC-treated cells (Figure 7A,B). These results indicate that IBC induced necroptosis in MDA-MB-231 cells could be mimicked by RIP3 upregulation.

### 2.5. IBC Induced Autophagy in MDA-MB-231 Cells

**Figure 8:**
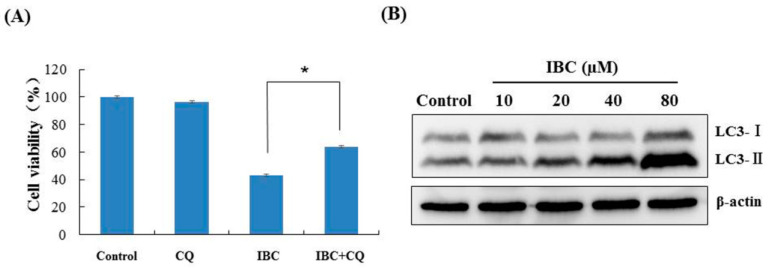
IBC induced autophagy in MDA-MB-231 cells. (**A**) Cell viability following treatment with IBC (40 μM) for 24 h with or without pre-treatment with the autophagy inhibitor CQ (20 μM) as measured by the MTT assay. (**B**) The expression of LC3 protein levels was analyzed by Western blotting. * *p* < 0.05 compared between the groups of IBC and IBC with CQ.

We also observed that IBC induced the morphological characteristics of typical autophagy at the same concentration (40 µM) and time (24 h) in MDA-MB-231 cells using electron microscopy (Figure 5D). Meanwhile, inhibition of autophagy with the autophagy inhibitor chloroquine (CQ) abolished the IBC cytotoxic activity of IBC in MDA-MB-231 cells (*p* < 0.05) (Figure 8A). Furthermore, we found that IBC substantially upregulated the LC3-II/I ratio, indicating the induction of autophagy (Figure 8B). These data suggest that IBC also induced autophagy in MDA-MB-231 cells.

### 2.6. Effect of IBC on Mitochondrial Function in MDA-MB-231 Cells 

**Figure 9:**
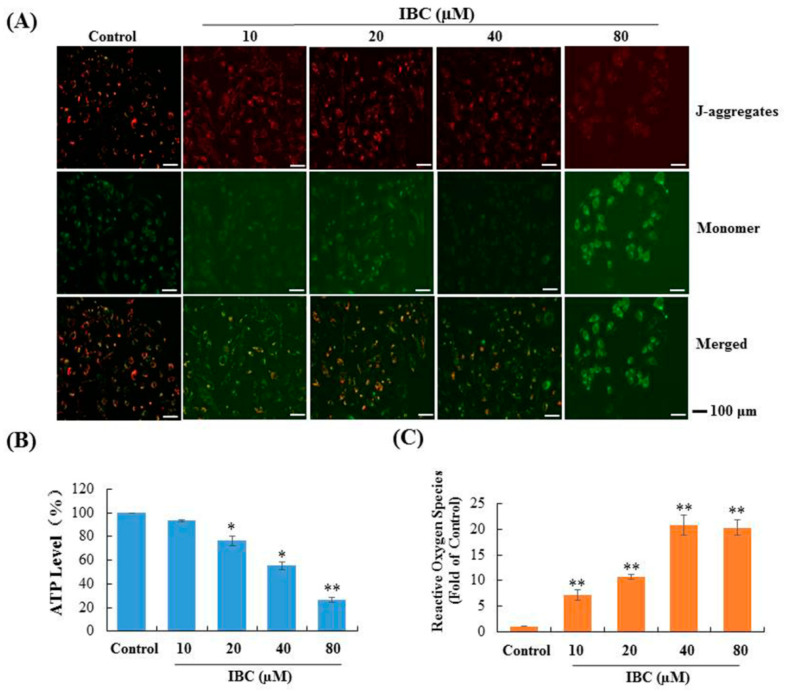
Effect of IBC on mitochondrial function in MDA-MB-231 cells. (**A**) Mitochondrial membrane potential was assessed by JC-1 staining and fluorescence microscopy. (**B**) Cellular ATP levels after treatment with various concentrations of IBC for 3 h. (**C**) IBC induced cellular ROS accumulation. * *p* < 0.05, ** *p* < 0.01 compared with the control.

A decline in mitochondrial membrane potential (MMP) is an important event during cell apoptosis or necroptosis. To explore whether IBC triggered multiple PCD is associated with mitochondrial function, we performed a fluorescent microscopy-based analysis by using the calcium indicator JC-1. As shown in Figure 9A, cells were treated at various concentrations (10, 20, 40, and 80 µM) of IBC, and a shift in JC-1 fluorescence from red to green was observed. Cellular ATP levels were significantly decreased to 93.3%, 76.6%, 55.1%, and 26.6% when treated with increasing concentrations at 10, 20, 40, and 80 µM of IBC, respectively (Figure 9B). In addition, we also observed that intracellular ROS levels gradually enhanced with increasing concentrations of IBC (*p* < 0.01) (Figure 9C). These results indicate that IBC induced multiple forms of MDA-MB-231 cell death that are associated with mitochondrial function.

### 2.7. In Vivo Anti-Tumor Efficacy of IBC

**Figure 10:**
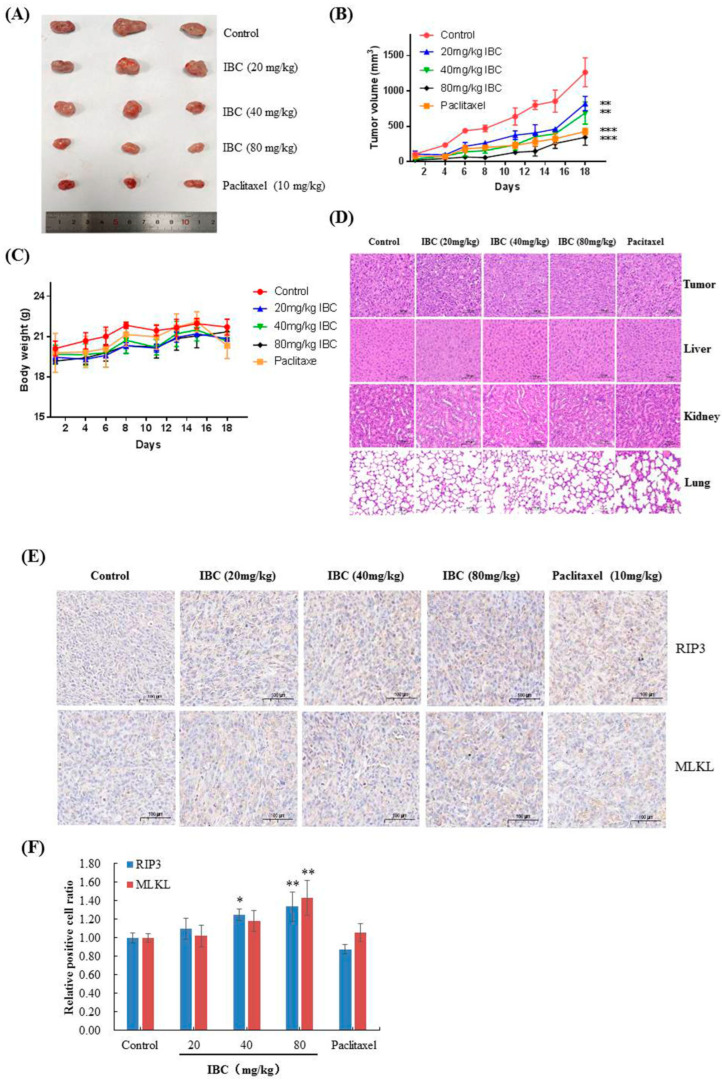
Anti-tumor efficacy of IBC in MDA-MB-231 cell xenografts in nude mice (*n* = 3). (**A**) Representative tumors from each treatment group. (**B**) Tumor volume of each treatment group. (**C**) Body weight changes of nude mice. (**D**) H and E-stained sections of the tumor, liver, kidney, and lung from the mice after treatment with increasing doses of IBC (20, 40, and 80 mg/kg). (**E**) Immunohistochemistry of RIP3 and MLKL. (**F**) Quantification of the immunohistochemistry. * *p* < 0.05, ** *p* < 0.01, and *** *p* < 0.001 compared with the control.

We used human triple-negative breast MDA-MB-231 cell xenograft models to assess the anti-breast cancer efficacy of IBC in vivo. Nude mice were intraperitoneally injected with IBC (20, 40, and 80 mg/kg) or vehicle for 18 days. At the end of the experiment, the mice were sacrificed, and the tumor volume and body weight were measured. IBC administration remarkably inhibited tumor growth in xenograft models in a dose-dependent manner (Figure 10A,B). When compared with that of the vehicle control group, 20, 40, and 80 mg/kg of IBC suppressed tumor growth by 35.0% (*p* < 0.01), 45.5% (*p* < 0.01), and 72.6% (*p* < 0.001), respectively. Paclitaxel, as a positive control, inhibited tumor growth by 66.1% in tumor volume (*p* < 0.001). All mice survived without appreciable body weight loss during the experiment (Figure 10C). Hematoxylin and eosin (H and E) staining of the liver, kidney, and lung tissue showed no obvious damage (Figure 10D). Immunohistochemical analyses showed that IBC induced a substantial increase in RIP3 and MLKL and activated the RIP1-RIP3-MLKL pathway in the MDA-MB-231 derived tumor (Figure 10E,F). These results were consistent with the data obtained at the cellular level and confirmed the anti-cancer properties of IBC in targeting RIP3-MLKL signaling pathways.

### 2.8. Molecular Docking of IBC on RIP3 

**Figure 11:**
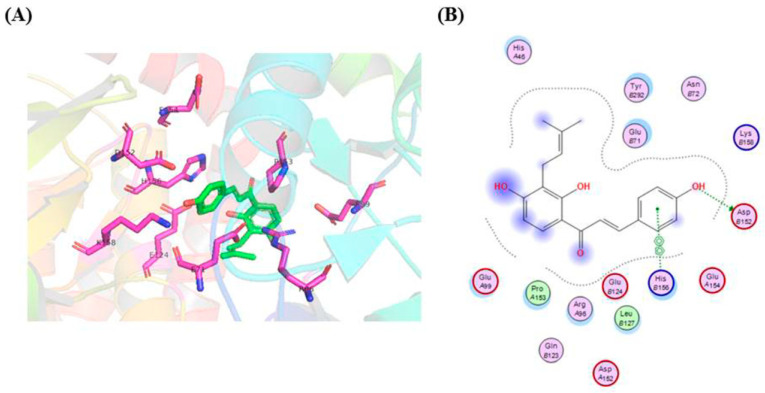
Molecular docking model of IBC in the active sites of the RIP3 protein. (**A**) Three-dimensional-binding models of IBC with RIP3. (**B**) A two-dimensional-binding model of IBC with RIP3.

In order to further explore the IBC and RIP3 interaction, we investigated whether the binding mode of IBC to RIP3 using a molecular docking. As shown in Figure 11, the phenolic hydroxyl moiety of the B ring of IBC formed hydrogen bonds with the Asp 152 residue. Moreover, a π-π interaction was formed between the B ring of IBC to occupy the His 156 residue of RIP3. The strong interaction between IBC and RIP3 supports the role of IBC as an RIP3-dependent necroptosis inducer.

## 3. Discussion

TNBC is one of the most fatal cancers and is more aggressive than other breast cancer types owing to lack of effective drugs and approved targets [4,17,30]. Although cytotoxic chemotherapy has been suggested for the treatment of TNBC, it eventually induces drug resistance and toxicity. In this study, we aimed to obtain effective drugs to treat TNBC from TCM or natural products and clarify their mechanism of action. Interestingly, we found that IBC (40 µM for 24 h) induced PCD in TNBC cells by triggering apoptosis, necroptosis, and autophagy. 

Apoptosis has emerged as an important therapeutic target that plays a substantial role in maintaining the balance between cell survival and death [31]. Our results showed for the first time that IBC induced apoptosis in MDA-MB-231 cells in a concentration-dependent manner. We found that IBC regulated apoptosis-related proteins (such as Akt, Bcl-2, and Bax) and induced apoptosis in MDA-MB-231 cells in the caspase-dependent manner. However, cancer cells are almost always deficient in the induction of apoptosis, resulting in anti-cancer drug resistance. Thus, the induction of multiple cell death signaling pathways in cancer cells may help overcome drug resistance or increase sensitivity to chemotherapy [32]. 

Herein, we found that apoptosis, necroptosis, and autophagy were responsible for the death inducing efficacy of IBC, whereas necroptosis might be more important for IBC induced cell death in MDA-MB-231 cells. Necroptosis is a recently discovered process that regulates the occurrence, development, metastasis, and drug resistance of cancers, and in particular, the immune response [33,34,35]. The state of RIP1 and RIP3 determines whether they function as molecules that inhibit or promote PCD [36]. Recent research has reported RIP3 as a key protein regulating the switch between cell survival and TNF-induced necroptosis and indicated that the complex containing RIP3 could function as a “necrosome” [29,37]. Our data showed that IBC treatment resulted in a substantial increase in the expression of RIP3, p-RIP3, and MLKL that are associated with the RIP3-MLKL signaling pathway. In the siRNA experiments, we demonstrated the importance of RIP3 in necroptosis. The data exhibited that knock-down of RIP3 expression inhibited IBC induced necroptosis and caused loss of the protective effect of Nec-1. 

Autophagy is a critical type of PCD and plays a dual role in the hallmarks of cancer [38]. Sometimes, the induction of autophagy significantly enhances chemotherapy sensitivity against cancer cells and reverses drug resistance. In contrast, combining standard anti-cancer drugs with an autophagy inhibitor could potentially accelerate cell death [39,40]. Our results showed that IBC also induced autophagy in MDA-MB-231 cells as evidenced by an increased LC3-II/I ratio.

Mitochondria serve as integrators of upstream death signaling that play a central role in cellular energy production and ROS accumulation [15]. High levels of intracellular ROS cause a disequilibrium between oxidative stress and antioxidants, leading to PCD [41]. RIP3 interacts with metabolic enzymes and is involved in the regulation of cellular energy metabolism and ROS accumulation during necroptosis [37]. In our study, IBC induced MDA-MB-231 cell death was closely associated with similar changes in mitochondrial function, mainly a decline in MMP, a decrease in ATP level, and the accumulation of large amounts of ROS. Therefore, our results suggest that IBC induced necroptosis in triple-negative breast MDA-MB-231 cells via the upregulation of the RIP3- MLKL-ROS pathway. 

## 4. Materials and Methods

### 4.1. Reagents and Chemicals 

Human breast cancer (MDA-MB-231 and MCF-7), hepatocellular carcinoma SMMC-7721, nasopharyngeal carcinoma CNE-2Z, and lung cancer H1975 cell lines were purchased from the cell bank of the Chinese Academy of Sciences (Shanghai, China). Dulbecco’s modified Eagle’s medium (DMEM), fetal bovine serum (FBS), and 0.25% trypsin were purchased from Gibco (New York, NY, USA). The BCA protein assay kit was purchased from Thermo Fisher Scientific (Atlanta, GA, USA). The annexin V/PI dual staining kit was purchased from BestBio Tech (Shanghai, China). The JC-1 assay kit, ATP assay kit, and ROS assay kit were purchased from Beyotime Institute of Biotechnology (Wuhan, China). The following antibodies were used: anti-Akt, anti-p-Akt-ser473, anti-Bcl-2, anti-Bax, anti-caspase-3, and anti-LC3 antibodies were purchased from Cell Signaling (Danvers, MA, USA); anti-RIP1, anti-RIP3, and anti-MLKL antibodies were purchased from Abcam (Cambridge, MA, UK); anti-GAPDH, anti-β-actin, goat anti-rabbit and goat anti-mouse secondary antibodies were purchased from BioSharp (Hefei, China). Dimethyl sulfoxide (DMSO), 3-(4,5-dimethylthiazol-2-yl)-2,5-diphenyl tetrazolium bromide (MTT), z-VAD-FMK, Nec-1, CQ, and 4,6-diamidino-2-phenyl indole (DAPI) were purchased from Sigma-Aldrich (St. Louis, MO, USA). IBC was isolated from the seeds of *P. corylifolia*, and its purity was higher than 96.5% as indicated by HPLC analysis [42]. Bavachin, isobavachin, bavachinin, isoliquiritigenin, shikonin, and paclitaxel were purchased from Yuanye Biological Technology (Shanghai, China).

### 4.2. Cell Culture 

The cells were cultured in DMEM supplemented with 10% FBS and 1% penicillin/streptomycin (Gibco, New York, NY, USA) in an atmosphere containing 5% CO_2_ at 37 °C. 

### 4.3. Cell Viability Assay

Cell viability was determined using the MTT assay. Cells were seeded in 96-well plates at a density of 6 × 10^3^ cells/well and treated with increasing concentrations of IBC (0, 5, 10, 20, 40, and 80 µM) for 24, 48, and 72 h. At the end of each time point, 10 µL of MTT (5 µg/mL) solution was added to each well and incubated at 37 °C in the dark. After 4 h, the medium was removed, and formazan was dissolved in 100 µL of DMSO for 10 min. Absorbance (A) was measured using a microplate spectrophotometer at 570 nm (Bio-Rad, Hercultes, CA, USA).

### 4.4. Colony-Formation Assay

Cells were seeded in 6-well plates at a density of 6 × 10^3^ cells per well and incubated overnight. When colonies formed, the medium was replaced with fresh medium (containing IBC) at different concentrations (0, 1, 2, and 4 µM) and incubated for 7 days. At the end of the incubation period, the cells were washed with phosphate buffered saline (PBS) solution, fixed with 4% paraformaldehyde solution for 10 min, stained with crystal violet solution for 10 min, and photographed. 

### 4.5. Flow Cytometry with Annexin V/PI Dual Staining

Cells were cultured overnight in 6-well plates at a density of 6 × 10^3^ cells per well. After adherence, the cells were treated with different concentrations (0, 10, 20, and 40 µM) of IBC and cultured for 24 h. At the end of the experiment, the cells were harvested from each well, washed with cold PBS solution, and stained with annexin V-FITC solution, followed by addition of PI staining solution and subsequent incubation at room temperature for 15 min in the dark. The percentage of cell death was analyzed using flow cytometry (BD Biosciences, Franklin Lakes, NJ, USA).

### 4.6. Fluorescence Staining

Cells were seeded in 12-well plates at a density of 1.5 × 10^5^ cells/well and treated with various concentrations (0, 10, 20, and 40 µM) of IBC. After being cultured for 24 h, the cells were fixed with 4% paraformaldehyde solution for 10 min, and then stained with DAPI solution for 10 min in the dark. Each well was washed with PBS and stained with annexin V-FITC solution, followed by the addition of PI staining solution and subsequent incubation at room temperature for 15 min. The fluorescent intensity of the cells was analyzed using a fluorescence microscope (Olympus, Japan).

### 4.7. Western Blotting 

Cells were seeded in 6-well plates at a density of 5 × 10^5^ cells/well. After treatment with various concentrations (0, 10, 20, 40, and 80 µM) of IBC, the cells were harvested, washed twice with PBS, and homogenized in RIPA lysis buffer for 30 min on ice. Protein concentration was measured using the BCA protein assay kit according to the manufacturer’s protocol. Equal amounts of proteins were loaded onto 10~12% SDS-PAGE and transferred to polyvinylidene difluoride membranes. The membrane was blocked using 5% skimmed milk and incubated with primary antibodies (Bcl-2, Bax, Akt, p-Akt-473, caspase-3, RIP1, RIP3, p-RIP3, MLKL, and LC3) overnight at 4°C, followed by the secondary antibodies at room temperature for 2 h. A chemiluminescence kit and gel imaging system were used to detect the protein bands (Bio-Rad, Hercultes, CA, USA). Anti- β-actin was used as an internal control.

### 4.8. Electron Microscopy

Cells were seeded in a 10-cm culture dish and cultured for 6 h with vehicle control and IBC (40 µM). After incubation, the cells were washed and fixed with 2.5% glutaraldehyde and stored at 4°C. Post-fixed specimens were sent to Servicebio (Wuhan, China) for subsequent processing and observation by transmission electron microscopy (TEM). The processed samples were imaged to observe the microscopic cellular structures as previously described. 

### 4.9. Immunofluorescence 

Cells were seeded in 12-well plates at a density of 1.2 × 10^5^ cells/well and treated with various concentrations (0, 10, 20, and 40 µM) of IBC. After being cultured for 24 h, the cells were fixed with paraformaldehyde for 10 min, permeabilized in 0.2% Triton X-100 for 10 min and incubated for 2 h in PBS solution containing 5% bovine serum albumin. Next, the cells were incubated overnight with RIP3 or MLKL antibodies at 4 °C and visualized using FITC-conjugated goat anti-rabbit IgG. Nuclei were stained with 2 µg/mL of DAPI in PBS, and images were observed using fluorescence microscopy (Olympus, Japan).

### 4.10. Small Interfering RNA (siRNA) Transfection

RIP3 siRNAs were obtained from Gene-Pharma (Shanghai, China) and were transiently transfected into MDA-MB-231 cells in 6-well plates using a lipofectamine 2000 reagent kit (Invitrogen, Carlsbad, CA, USA). After 24 h of transfection, the cells were collected for Western blotting analysis as described earlier. The siRNA sequences used for experiments with human RIP3 were as follows: positive control sense, 5′-UGA CCU CAA CUA CAU GGU UTT-3′ and antisense, 5′-AAC CAU GUA GUU GAG GUC ATT-3′; negative control sense, 5′-UUC UCC GAA CGU GUC ACG UTT-3′ and antisense, 5′-ACG UGA CAC GUU CGG AGA ATT-3′; RIP3 homo-478 sense, 5′-CCG GCU CUG GUG ACU AAA UTT-3′ and antisense, 5′-AUU UAG UCA CCA GAG CCG GTT-3′; RIP3 homo-570 sense, 5′-GCU GAA AGA AGU GGU GCU UTT-3′ and antisense, 5′-AAG CAC CAC UUC UUU CAG CTT-3′; and RIP3 homo-775 sense, 5′-GAA CUG UUU GUU AAC GUA ATT-3′ and antisense, 5′-UUA CGU UAA CAA ACA GUU CTT-3′. 

### 4.11. Detection of MMP with JC-1 

Cells were seeded in 6-well plates at a density of 2 × 10^5^ cells/well. After adherence, the cells were treated with different concentrations (0, 10, 20, 40, and 80 µM) of IBC. After 24 h, JC-1 staining solution (1 mL) and fresh medium (1 mL) were added to each well and then mixed and incubated for 20 min at 37°C. Afterwards, the supernatant was aspirated from the cell, followed by washing twice with cold JC-1 staining buffer. Following the manufacturer’s protocol, the cells were observed and imaged using fluorescence microscopy (Olympus, Japan).

### 4.12. Detection of Intracellular ATP Levels 

Cells were seeded in 12-well plates at a density of 2 × 10^5^ cells/well and treated with various concentrations (0, 10, 20, 40, and 80 µM) of IBC for 5 h. At the end of the experiment, the cells were washed, lysed with RIPA buffer, and the lysate was centrifuged at 12,000 rpm for 5 min. To detect intracellular ATP levels, we used a luminometric-based ATP assay kit according to the manufacturer’s protocol. The signal was measured using a luminoskan luminometer (Thermo Scientific, Atlanta, GA, USA).

### 4.13. Detection of ROS 

Cells were seeded in 12-well plates at a density of 2 × 10^5^ cells/well and treated with various concentrations (0, 10, 20, 40, and 80 µM) of IBC for 3 h. After centrifugation at 1500 rpm for 10 min, the supernatant was removed, followed by the addition of 500 µL of DCFH-DA solution at 37℃. Intracellular ROS levels were analyzed using flow cytometry (BD Biosciences, Franklin Lakes, NJ, USA). 

### 4.14. In Vivo Experiments

BALB/c (nu/nu) female athymic mice, aged 4–5 weeks, were purchased from Shanghai Slac Laboratory (China). All animal experiments were approved by the Animal Care and Use Committee of Bengbu Medical College. Human breast cancer MDA-MB-231 cells (5 × 10^6^ cells per mouse) were subcutaneously injected to induce tumor formation. When the average tumor volume reached approximately 100 mm^3^, the mice were randomly divided into five groups (three mice per group), and each group were treated with 0.2 mL of saline, IBC (20 mg/kg), IBC (40 mg/kg), IBC (80 mg/kg), or paclitaxel (10 mg/kg). The mice were intraperitoneally injected at 1, 4, 6, 8, 11, 13, 15, and 18 days, and the tumor volume and body weight were monitored before each injection. Tumor volumes were measured as [lengh × width^2^]/2 (means ± SD). In addition, the solid tumor, liver, kidney, and lung were removed and stored in 4% formalin solution and stained with H and E.

### 4.15. Immunohistochemistry

Briefly, paraffin sections were prepared according to the following steps: dewaxing, endogenous peroxidase blockade using hydrogen peroxide (H_2_O_2_), serum blockade before being treated with the anti-RIP3 and anti-MLKL antibodies (1:100) at 4 ℃, and biotin-conjugated secondary antibody (1:100) at 37 ℃. Visualization was performed using DAB, followed by dehydration, transparency, and tissue mounting. The number of positive targets were counted using a microscope at 400× magnification and the positive area (brown-yellow area) of the experimental group was compared with the control group for statistical analysis.

### 4.16. Molecular Docking

PyRx was used for molecular modeling and docking studies. The structure of IBC bound to RIP3 (PDB code: 4M66) was used for modeling experiments using Auto Dock Vina. PyMOL was used for further visualization, figure preparation, and conformational analyses.

### 4.17. Statistical Analysis

SPSS v.16.0 software (SPSS Inc., Chicago, IL, USA) was used for statistical analysis. Data are presented as the means ± SD from three independent experiments. Statistical comparisons between groups were analyzed using one-way ANOVA followed by the student’s *t*-test. * *p* < 0.05, ** *p* < 0.01, and *** *p* < 0.001 were considered statistically significant.

## 5. Conclusions

In summary, IBC exhibited potent anti-cancer properties in triple-negative breast MDA-MB-231 cells with low toxicity. Importantly, our observations revealed that IBC induced multiple cell death in MDA-MB-231 cells by triggering apoptosis, necroptosis, and autophagy. In particular, IBC induced necroptosis by upregulating RIP3-MLKL signaling and ROS accumulation. Therefore, IBC may be a potential anti-cancer agent for the discovery and development of anti-TNBC drugs. 

## Figures and Tables

**Table 1:** Cytotoxic activity (IC_50_, 72 h) for IBC and its analogs against five cancer cell lines.

Compounds	MDA-MB-231	MCF-7	SMMC-7721	CNE-2Z	H1975
IBC	8.53	>25.0	12.95	>25.0	14.54
isoliquiritigenin	>25.0	16.26	>25.0	23.40	>25.0
isobavachin	>25.0	>25.0	>25.0	>25.0	>25.0
bavachin	16.95	>25.0	>25.0	23.51	>25.0
bavachinin	22.08	>25.0	>25.0	>25.0	>25.0
shikonin ^a^	1.27	1.35	0.59	3.87	3.18

^a^ positive control.

## Data Availability

Data are contained within the article.

## References

[1] Siegel R.L., Miller K.D., Jemal A. (2020). Cancer statistics, 2020. CA Cancer J. Clin..

[2] Anders C.K., Carey L.A. (2009). Biology, metastatic patterns, and treatment of patients with triple-negative breast cancer. Clin. Breast Cancer.

[3] Mathe A., Wong-Brown M., Morten B., Forbes J.F., Braye S.G., Avery-Kiejda K.A., Scott R.J. (2015). Novel genes associated with lymph node metastasis in triple negative breast cancer. Sci. Rep..

[4] Jiang Y.Z., Ma D., Suo C., Shi J., Xue M., Hu X., Xiao Y., Yu K.D., Liu Y.R., Yu Y. (2019). Genomic and transcriptomic landscape of triple-negative breast cancers: Subtypes and treatment strategies. Cancer Cell.

[5] Gibellini L., Moro L. (2021). Programmed cell death in health and disease. Cells.

[6] Pommier Y., Sordet O., Antony S., Hayward B.L., Kohn K.W. (2004). Apoptosis defects and chemotherapy resistance: Molecular interaction maps and networks. Oncogene.

[7] Manne R.K., Agrawal Y., Malonia S.K., Banday S., Edachery S., Patel A., Kumar A., Shetty P., Santra M.K. (2021). FBXL20 promotes breast cancer malignancy by inhibiting apoptosis through degradation of PUMA and BAX. J. Biol. Chem..

[8] Hu X., Han W., Li L. (2007). Targeting the weak point of cancer by induction of necroptosis. Autophagy.

[9] Degterev A., Huang Z., Boyce M., Li Y., Jagtap P., Mizushima N., Cuny G.D., Mitchison T.J., Moskowitz M.A., Yuan J. (2005). Chemical inhibitor of nonapoptotic cell death with therapeutic potential for ischemic brain injury. Nat. Chem. Biol..

[10] Vanlangenakker N., Vanden B.T., Vandenabeele P. (2012). Many stimuli pull the necrotic trigger, an overview. Cell Death Differ..

[11] Hitomi J., Christofferson D.E., Ng A., Yao J., Degterev A., Xavier R., Yuan J. (2008). Identification of a molecular signaling network that regulates a cellular necrotic cell death pathway. Cell.

[12] Wu Y., Dong G., Sheng C. (2020). Targeting necroptosis in anticancer therapy: Mechanisms and modulators. Acta. Pharm. Sin. B.

[13] Grassilli E., Narloch R., Federzoni E., Lanzano L., Pisano F., Giovannoni R., Romano G., Masiero L., Leone B.E., Bonin S. (2013). Inhibition of GSK3β bypass drug resistance of p53-null colon carcinomas by enabling necroptosis in response to chemotherapy. Clin. Cancer Res..

[14] Han W., Li L., Qiu S., Lu Q., Pan Q., Gu Y., Luo J., Hu X. (2007). Shikonin circumvents cancer drug resistance by induction of a necroptotic death. Mol. Cancer Ther..

[15] Li L., Han W., Gu Y., Qiu S., Lu Q., Jin J., Luo J., Hu X. (2007). Honokiol induces a necrotic cell death through the mitochondrial permeability transition pore. Cancer Res..

[16] Wu M., Jiang Z., Duan H., Sun L., Zhang S., Chen M., Wang Y., Gao Q., Song Y., Zhu X. (2013). Deoxypodophyllotoxin triggers necroptosis in human non-small cell lung cancer NCI-H460 cells. Biomed. Pharm..

[17] Won K.A., Spruck C. (2020). Triple-negative breast cancer therapy: Current and future perspectives (Review). Int. J. Oncol..

[18] Yang Z., Zhang Q., Wu L., Zhu J., Cao Y., Gao X. (2021). The signaling pathways and targets of traditional Chinese medicine and natural medicine in triple-negative breast cancer. J. Ethnopharmacol..

[19] Liu Y., Yang S., Wang K., Lu J., Bao X., Wang R., Qiu Y., Wang T., Yu H. (2020). Cellular senescence and cancer: Focusing on traditional Chinese medicine and natural products. Cell Prolif..

[20] Bhalla V.X., Nayak U.R., Dev S. (1968). Some new flavonoids from *Psoralea corylifolia*. Tetrahedron Lett..

[21] Nishimura R., Tabata K., Arakawa M., Ito Y., Kimura Y., Akihisa H., Nagai H., Sakuma A., Kohno H., Suzuki T. (2007). Isobavachalcone constituent of Angeltca ketsket induces apoptosis in neuroblastoma. Biol. Pharm. Bull..

[22] Mbaveng A.T., Ngameni B., Kuete V., Simo I.K., Ambassa P., Roy R., Bezabih M., Etoa F.X., Ngadjui B.T., Abegaz B.M. (2008). Antimicrobial activity of the crude extracts and five flavonoids from the twigs of *Dorstenia barteri* (Moraceae). J. Ethnopharmacol..

[23] Lim S.H., Ha T.Y., Ahn J., Kim S. (2011). Estrogenic activities of *Psoralea corylifolia* L. seed extracts and main constituents. Phytomedicine.

[24] Jing H., Zhou X., Dong X., Cao J., Zhu H., Lou J., Hu Y., He Q., Yang B. (2010). Abrogation of Akt signaling by isobavachalcone contributes to its anti-proliferative effects towards human cancer cells. Cancer Lett..

[25] He H., Wang C., Liu G., Ma H., Jiang M., Li P., Lu Q., Li L., Qi H. (2021). Isobavachalcone inhibits acute myeloid leukemia: Potential role for ROS-dependent mitochondrial apoptosis and differentiation. Phytother. Res..

[26] Li B., Xu N., Wan Z., Ma L., Li H., Cai W., Chen X., Huang Z., He Z. (2019). Isobavachalcone exerts anti-proliferative and proapoptotic effects on human liver cancer cells by targeting the ERKs/RSK2 signaling pathway. Oncol. Rep..

[27] Shi J., Chen Y., Chen W., Tang C., Zhang H., Chen Y., Yang X., Xu Z., Wei J., Chen J. (2018). Isobavachalcone snesitizes cells to E2-induced paclitaxel resistance by down- regulating CD44 expression in ER+ breast cancer cells. J. Cell. Mol. Med..

[28] Zhang Y., Gao M., Zhu M., Li H., Ma T., Wu C. (2022). Isobavachalcone induces cell death through multiple pathways in human breast cancer MCF-7 cells. J. South Med. Univ..

[29] Moriwaki K., Chan F.K. (2013). RIP3: A molecular switch for necrosis and inflammation. Genes Dev..

[30] Gupta N., Gaikwad S., Kaushik I., Wright S.E., Markiewski M.M., Srivastava S.K. (2021). Atovaquone suppresses triple-negative breast tumor growth by reducing immune-suppressive cells. Int. J. Mol. Sci..

[31] Chung C. (2018). Restoring the switch for cancer cell death: Targeting the apoptosis signaling pathway. Am. J. Health Syst. Pharm..

[32] Urtishak K.A., Edwards A.Y., Wang L.S., Hudome A., Robinson B.W., Barrett J.S., Cao K., Cory L., Moore J.S., Bantly A.D. (2013). Potent obatoclax cytotoxicity and activation of triple death mode killing across infant acute lymphoblastic leukemia. Blood.

[33] Woo Y., Lee H.J., Jung Y.M., Jung Y.J. (2020). Regulated necrotic cell death in alternative tumor therapeutic strategies. Cells.

[34] Su Z., Yang Z., Xu Y., Chen Y., Yu Q. (2015). Apoptosis, autophagy, necroptosis, and cancer metastasis. Mol. Cancer.

[35] Snyder A.G., Hubbard N.W., Messmer M.N., Kofman S.B., Hagan C.E., Orozco S.L., Chiang K., Daniels B.P., Baker D., Oberst A. (2019). Intratumoral activation of the necroptotic pathway components RIPK1 and RIPK3 potentiates antitumor immunity. Sci. Immunol..

[36] Silke J., Richard J.A., Gerlic M. (2015). The diverse role of RIP kinases in necroptosis and inflammation. Nat. Immunol..

[37] Zhang D.W., Shao J., Lin J., Zhang N., Lu B.J., Lin S.C., Dong M.Q., Han J. (2009). RIP3, an energy metabolism regulator that switches TNF-induced cell death from apoptosis to necrosis. Science.

[38] Singh S.S., Vats S., Chia A.Y., Tan T.Z., Deng S., Ong M.S., Arfuso F., Yap C.T., Goh B.C., Sethi G. (2018). Dual role of autophagy in hallmarks of cancer. Oncogene.

[39] Sun X., Yan P., Zou C., Wong Y.K., Shu Y., Lee Y.M., Zhang C., Yang N.D., Wang J., Zhang J. (2019). Targeting autophagy enhances the anticancer effect of artemisinin and its derivatives. Med. Res. Rev..

[40] Cocco S., Leone A., Piezzo M., Caputo R., Lauro V.D., Rella F.D., Fusco G., Capozzi M., Gioia G.D., Budillon A. (2020). Targeting autophagy in breast cancer. Int. J. Mol. Sci..

[41] Galadari S., Rahman A., Pallichankandy S., Thayyullathil F. (2017). Reactive oxygen species and cancer paradox: To promote or to suppress?. Free Radic. Biol. Med..

[42] Ma T., Nie L.J., Li H.M., Huo Q., Zhang Y.X., Wu C.Z. (2015). Determination of isobavachalcone in rat plasma by LC-MS/MS and its application to a pharmacokinetic study. J. Pharm. Biomed. Anal..

